# Oligometastases from prostate cancer: local treatment with stereotactic body radiotherapy (SBRT)

**DOI:** 10.1186/s12885-017-3341-2

**Published:** 2017-05-22

**Authors:** Gregor Habl, Christoph Straube, Kilian Schiller, Marciana Nona Duma, Markus Oechsner, Kerstin A. Kessel, Matthias Eiber, Markus Schwaiger, Hubert Kübler, Jürgen E. Gschwend, Stephanie E. Combs

**Affiliations:** 1 Department of Radiation Oncology, Technical University of Munich (TUM), Ismaninger Strasse 22, 81675 Munich, Germany; 2 Zentrum für Stereotaxie und personalisierte Hochpräzisionsstrahlentherapie (StereotakTUM), Technische Universität München (TUM), Munich, Germany; 3 Institute of Innovative Radiotherapy (iRT), Department of Radiation Sciences (DRS), Helmholtz Zentrum München, Neuherberg, Germany; 4 Department of Nuclear Medicine, Technical University Munich (TUM), Munich, Germany; 5 Department of Urology, Technical University Munich (TUM), Munich, Germany; 6 Department of Urology, University of Würzburg, Würzburg, Germany

**Keywords:** Prostate cancer, Oligometastases, Individualized radiotherapy, SBRT, PSMA-PET, PSA

## Abstract

**Background:**

The impact of local tumor ablative therapy in oligometastasized prostate cancer (PC) is still under debate. To gain data for this approach, we evaluated oligometastasized PC patients receiving stereotactic body radiotherapy (SBRT) to bone metastases.

**Methods:**

In this retrospective study, 15 oligometastasized PC patients with a total of 20 bone metastases were evaluated regarding biochemical progression-free survival (PSA-PFS), time to initiation of ADT, and local control rate (LCR). Three patients received concomitant androgen deprivation therapy (ADT).

**Results:**

The median follow-up after RT was 22.5 months (range 7.0–53.7 months). The median PSA-PFS was 6.9 months (range 1.1–28.4 months). All patients showing a decrease of PSA level after RT of at least factor 10 reveal a PSA-PFS of >12 months. Median PSA-PFS of this sub-group was 23.1 months (range 12.1–28.4 months). Local PFS (LPFS) after 2 years was 100%. One patient developed a local failure after 28.4 months. Median distant PFS (DPFS) was 7.36 months (range 1.74–54.34 months). The time to initiation of ADT in patients treated without ADT was 9.3 months (range 2.6–36.1 months). In all patients, the time to intensification of systemic therapy or the time to initiation of ADT increased from 9.3 to 12.3 months (range 2.6–36.1 months). Gleason-Score, ADT or the localization of metastasis had no impact on PFS or time to intensification of systemic therapy. No SBRT related acute or late toxicities were observed.

**Conclusion:**

Our study shows that SBRT of bone metastases is a highly effective therapy with an excellent risk-benefit profile. However, PFS was limited due to a high distant failure rate implying the difficulty for patient selection for this oligometastatic concept. SBRT offers high local cancer control rates in bone oligometastases of PC and should be evaluated with the aim of curation or to delay modification of systemic treatment.

## Background

Therapy concepts in prostate cancer (PC) exist for local, advanced locoregional and metastatic diseases, but the situation for non-symptomatic oligometastatic disease remains unclear [1]. A specific subgroup of patients presents with few lesions, whereas others develop rapid progression in multiple sites. For the first group, the term “oligometastases” is used to define five or less metastatic lesions per population. Therapeutically recommended are either a surveillance strategy in the absence of symptoms and slow rise of PSA level, or palliative androgen suppression in case of rapid PSA level rise with a doubling of PSA in less than 3 months or when symptomatic metastasis occur [2]. Because androgen deprivation therapy (ADT) is associated with a marked reduction in quality of life, current NCCN guidelines version 3.2016 suggest a surveillance strategy in case of asymptomatic metastases. Furthermore, randomized trials for metastatic PC have shown a superiority of early docetaxel-based chemotherapy combined with ADT androgen suppression [3, 4]. However, a not negligible number of grade 3–4 toxicities developed in patients undergoing this aggressive combined regimen. As various retrospective studies suggest that the occurrence of isolated metastases could potentially identify a subgroup of patients with a better prognosis despite systemic spread, this subgroup of patients might present a population that could benefit from a regional treatment [5]. The distinction between manifest polymetastases and oligometastases has been a central part of the oncological treatment strategy for years, for example in breast cancer, melanomas, sarcomas, renal cell and colorectal carcinomas [6–9]. The local treatment of liver metastases in oligometastatic colorectal cancer, for instance, leads to a significant life-prolongation, as does the local treatment of sarcoma metastases deriving from different localizations [9].

Retrospective studies discussing the treatment of solitary or few metastases in patients with PC suggest an improvement of biochemical and clinical relapse-free survival, especially in lymph node and bone metastases [10].

The boundary between oligometastatic and polymetastatic disease varies from three to five metastases [1, 5, 11, 12]. The published data on local metastasis-directed therapy describe a progression-free survival (PFS) between 12 and 24 months [1]. The key to successful local treatment of oligometastasized patients is precise imaging. For prostate cancer, almost all published studies thus far used ^11^C–choline PET for staging [1]. Particularly for the detection of very small lymph nodes, this technique has a low sensitivity of <10% [13, 14]. Hence, the probability of under-staging is enormous. In addition, a PSA level of at least 1–2 ng/ml is currently recommended in order to have a good chance of detecting tumor affected regions. ^68^Ga-Prostate specific membrane antigen (PSMA)-PET seems to be significantly more sensitive and specific [15, 16]. Here, significant findings are possible with a PSA level starting as low as 0.2 ng/ml. Afshar-Oromieh et al. published sensitivity levels of 76.6% and very high specificity levels of up to 100% [17]. Only recently, we have shown a highly significant benefit of ^68^Ga-PSMA for staging prior to radiation therapy [18]. Currently, there are no prospective data for the treatment of oligometastatic PC using PSMA PET for staging. However, a retrospective case collection of Demirkol et al. describes significant therapy changes due to the high diagnostic value of ^68^Ga-PSMA-PET [19].

Since there are currently no evident treatment recommendations for the subgroup of oligometastasized PC patients due to limited knowledge about the importance of aggressive local treatments, different therapeutic strategies are currently in use, including continuous ADT, intermittent ADT, high-dose radiotherapy (RT) with or without ADT, surgery and surveillance strategies. This retrospective study investigates PC patients treated for oligometastatic disease with high-dose RT to the metastatic lesions analyzing biochemical PFS (PSA-PFS), the time to initiation of ADT, the local control rate (LCR) and distant PFS (DPFS).

## Methods

In the framework of the STEREOTAKTUM, we offer stereotactic body radiotherapy (SBRT) and other highly-advanced treatments in tight interdisciplinary connection with all neighboring oncology disciplines including radiology and nuclear medicine imaging. For oligometastasized PC patients, SBRT was offered to patients with a maximum of two bone metastases based on PSA-values in connection with MRI and PET-imaging. Out of 205 patients with bone metastases of PC who received radiotherapy between March 2012 and April 2016. Only 15 patients with a total of 20 bone metastases cases underwent tumor ablative SBRT with curative intention, all others were treated with a palliative dose. The study was approved by the local ethics committee at the Medical Faculty of the Technische Universität München (TUM), vote number 257/16 S.

### Patients’ characteristics

All but one patient received primary surgery between 1994 and 2012. One patient received primary hormone-chemotherapy and surgery followed in 2016. The median time between primary diagnosis and SBRT was 55.4 months (range 6.7–208.3 months). Eleven patients had single metastases, two patients had two synchronous metastases, one patient had two metachronous metastases in a one-year interval and another patient had three metachronous metastases in a two-year interval between metastasis one and two, and 10 months between metastasis two and three. All patients were staged with PET-CT, 13 metastases (65%) were found and staged with ^68^Ga-PSMA-PET, four metastases (20%) with ^11^C–choline PET imaging, and three (15%) metastases were seen and staged with both, respectively. Patient characteristics and localization of bone metastases are shown in Table 1. Median age at start of RT was 69 years (range 55–76 years). The median PSA level before RT was 1.99 ng/ml (range 0.44–11.7 ng/ml). Primary tumors were classified according to Gleason Score as well as the novel prognostic Gleason grade grouping of the 2014 Chicago grading meeting [20, 21]. According to the D’Amico criteria, all patients were scored as high-risk due to PSA level or T-stage.

**Table 1:** Patients’ characteristics and localization of treated oligometastases

Age (years)	72 (range 56–78)
Concomitant ADT yes/no	3/12
PSA before SBRT	1.99 (range 0.44–11.7)
Initial Gleason Score^a^	
6 (group1)	1
7a (group 2)	5
7b (group 3)	0
8 (group 4)	4
9 (group 5)	4
Initial PSA (ng/ml) before primary treatment	17.0 (range 4.3–57.2)
Initial N0/N1	9/6
Localization of bone metastases (*n* = 20)	
Pelvis	6
Spine	8
Rib	5
Scapula	1

^a^in one patient no GS was available

The median time between diagnosis of PC and diagnosis of the oligometastatic disease was 4.6 years (range 0–12 years). One patient had a synchronous bone metastasis at primary staging explaining the value zero at the range level. All other patients had metachronous metastases. The median time between diagnosis of oligometastases and start of RT was 2.1 months (range 0.7–6.9 months). Three patients received concomitant ADT. Since this is a retrospective study, in these three patients, ADT was prescribed by their urologist prior to initial presentation at our clinic. In one patient, ADT was started to bridge the time to local therapy, in another, SBRT was indicated after primary hormone-chemotherapy, and another patient showed a PSA elevation during ADT indicating hormonal resistance.

### Radiotherapy treatment planning

High-dose RT was conducted in an SBRT-set-up. Radiation was applied as multi-field 3D–RT in six cases (five rib metastases and one pubic metastasis), as volumetric modulated arc therapy (VMAT) in seven cases and in two cases as TomoTherapy. One patient received a combined VMAT/3D plan as seen in Table 2. Patients were treated with a Varian Clinac Trilogy linear accelerator equipped with a 120 HD MLC (Varian Medical Systems, USA) or received TomoTherapy (Accuray, USA). CTV included the whole vertebra without the transverse and spinous process in cases with bone metastases to the vertebra body or pedicle. CTV included the transverse or spinous process if one of these structures were affected. CTV of bone metastases of the pelvis, ribs, and scapula included the GTV plus a safety margin of 1–2 cm depending on the localization and neighboring structures. We often conducted 4D–CT in the planning period to eliminate breathing disparities.

**Table 2:** Patients’ characteristics and treatment

Patient	Gleason-Score	Concomitant ADT	Time between occurrence of metastasis and first diagnosis (years)	Tumor site	RT dose (Gy)	Prescribed dose in percentage	EQD2 (Gy) α/β = 2; α/β = 3	RT technique	Time between progress and end of RT (months)	PSA before RT (ng/ml)	PSA nadir (ng/ml)	PSA in progress (ng/ml)
1	8	No	3.1	Lumbar vertebra 2	5 × 6 (PTV), 5 × 8 (SIB)	Median	100; 88	VMAT	25.3	2.3	0.1	7.0
		No	3.1	8th rib right	5 × 6.5	60%	173; 150	3D		2.3	0.1	7.0
2	8	Yes	2.8	Scapula left	5 × 6 PTV (GTV + asym. Safety margin) 5 × 8 SIB (GTV + 5 mm)	30Gy-isodose encloses 95% of PTV, SIB 95%	109; 96	VMAT	9.3	1.9	3.3 (x)	160
3	7	No	2.4	Pubic bone	5 × 6 PTV (GTV + 2 cm + 5 mm) 5 × 8 SIB (GTV + 5 mm) adapted to the bone margin	30Gy-isodose encloses 85% of PTV, SIB median	100; 88	VMAT	10.5	0.4	0.3	0.5
		No	3.4	Iliac bone	5 × 6 PTV (GTV + 2 cm + 5 mm) 5 × 8 SIB (GTV + 5 mm) adapted to the bone margin	30Gy-isodose encloses PTV, SIB median	100; 88	VMAT	12.5	0.5	0.05	(y)
4	8	No	5.3	Cervical vertebra 5	5 × 5 (whole vertebra without spinal canal) 5 × 8 (SIB)	95%	109; 96	VMAT +3D	1.1	2.9	4.3 (x)	6.2
5	8	No	0.3	Thoracic vertebra 9, transverse process left	5 × 5 (whole vertebra without spinal canal) 5 × 8 SIB (GTV)	30Gy-isodose encloses 70% of PTV, SIB median	100; 88	Tomotherapy	3.4	11.7	6.8	12
6	7	No	4.3	Lumbar vetrebra 4	5 × 6 (whole vertebra without spinal canal) 5 × 8 SIB (GTV + 5 mm)	30Gy-isodose encloses 60% of PTV, SIB median	100; 88	VMAT	6.2	0.9	0.9	10.7
7	9	No	4.6	10th rib right	5 × 5.4	60%	124; 108	3D	6.9	4.7	3.9	6.9
		No	4.6	Iliac bone left	5 × 6 PTV (GTV + asym. Safety margin) 5 × 8 SIB (GTV)	30Gy-isodose encloses 95% of PTV, 40 Gy-isodose encloses SIB		3D	6.9	4.7	3.9	6.9
8	7	Yes	6.0	Pubis bone right	5 × 7	60%	200; 171	3D	6.6	2.2	1.8	2.4
9	9	No	5.3	2nd rib left	5 × 6	60%	150; 130	3D	N	1.2	0.0	(y)
10	6	No	10.4	5th rib left	5 × 6	60%	150; 130	3D	1.7	2.0	(z)	4.3
11	7	No	4.3	Thoracic vertebra 7	5 × 5 (whole vertebra without spinal canal) 5 × 8 (SIB)	Median	100; 88	TomoTherapy	28.4	2.7	0.17	0.8
12	9	No	0.9	Pubic bone right	5 × 6	60%	150; 130	VMAT	6.9	0.9	1.3 (x)	4.1
13	7	Yes	0.0	Lumbar vertebra 4	5 × 5 (whole vertebra without spinal canal) 5 × 7 (SIB)	95%	86, 77	VMAT	N	15.0 (iPSA)	n.a. (w)	n.a.
14	G3	No	17.2	4th rib left	5 × 7	60%		3D	12.1	1.5	0.3	3.4
15	9	No	9.1	Acetabulum left	5 × 6 (Acetabulum dorsal) 5 × 7 (GTV + asym. Safety margin)	95%	86; 77	VMAT	23.1	3.9	0.3	0.9
		No	11.2	Thoracic vertebra 4, Spinous process	5 × 5 (Spinous process) 5 × 7 (GTV)	95%	86; 77	VMAT	3.6	0.9	0.19	0.8
		No	12.0	Thoracic vertebra 5	5 × 7 Gy, GTV + 2 mm (due to pre-RT)	median	79; 70	3D	2.1	0.8	(z)	n.a.

(x) = shows no nadir, PSA level increases directly after RT

(y) = no progress until now

(z) = ADT was started 4 weeks after RT, so nadir was not available

(w) = patient started concomitant hormone-chemotherapy, surgery of the primary follows

Delivered doses to spinal bone metastases were 25 or 30 Gy in 5 fractions for the whole vertebra with a simultaneously integrated boost (SIB) to the gross tumor volume (GTV) of 35 or 40 Gy (corresponding to the 60% (95%) isodose). Correspondent EQD2 doses are specified in Table 2. For TomoTherapy plans, the dose was normalized so that the 60% isodose covers 100% of the PTV. Pelvic bone metastases were treated unequally with 25, 30 and 35 Gy in 5 fractions or with the SIB concept mentioned above with 30 Gy to a larger volume and a SIB to the GTV of 40 Gy in 5 fractions (prescribed to the 60% isodose). Rib metastases were treated with total doses between 27 and 35 Gy in 5 fractions (prescribed to the 60% isodose). The bone metastasis of the scapula was treated with 30 Gy to a larger volume and 40 Gy to the GTV (prescribed to the 60% isodose). All information concerning the radiation treatment can be found in Table 2.

### Follow-up and statistics

All patients were included in a thorough clinical follow-up including PSA-evaluations, clinical assessment as well as MRI or PET-imaging. The first follow-up visit is scheduled 6 to 8 weeks after SBRT and every 6 months subsequently. Biochemical recurrence was defined as (i) a rising PSA level out of post-RT nadir to ≥0.2 ng/ml plus another higher value, or (ii) a decreased serum PSA, but above 0.2 ng/mL and increased again, or (iii) a continually rising post-RT PSA level, or (iv) clinical progression.

Distant PFS (DPFS) was defined as the absence of a new metastatic lesion. Local PFS (LPFS) was defined as tumor progression within the irradiated planning target volume. Patient data is collected in a center-specific database (MiRO-database) including all relevant information.

Statistical calculations were performed using SPSS Statistics v23 (IBM, USA). Survival analyses (PSA-PFS, LPFS, DPFS and time to ADT) were based on the Kaplan-Meier method. Calculations were done from the end of SBRT. For analyses of different groups, we used the log-rank test. A *p*-value ≤0.05 was considered as statistically significant.

## Results

### Tolerability of SBRT

SBRT was well tolerated and could be completed as planned in all patients. No specific acute or late toxicities occurred. Of note, we did not observe any bone fractures in patients treated for rib lesions or other osseous targets.

### Progression-free survival and course of PSA-levels

**Fig. 1:**
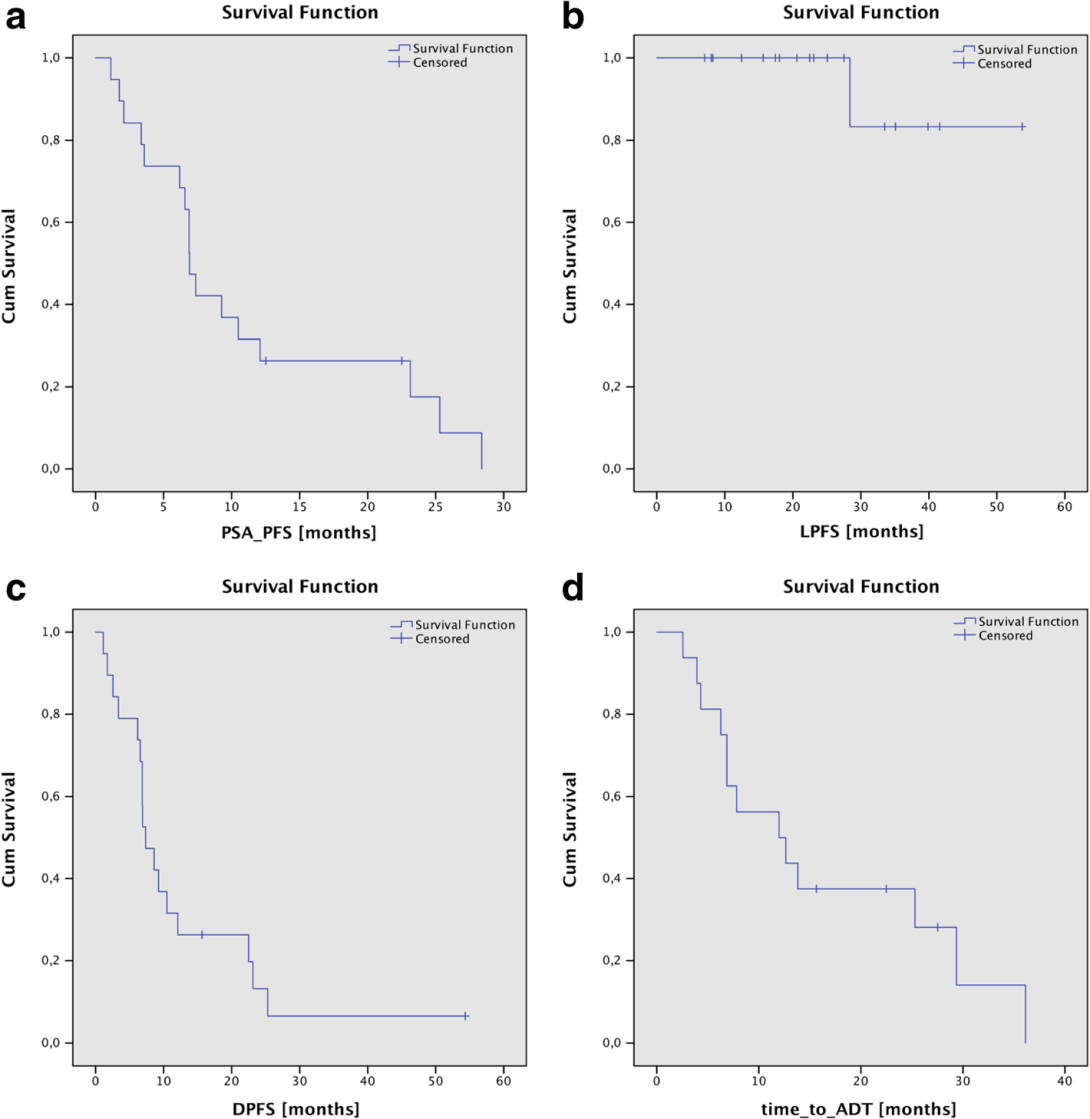
PSA-PFS (**a**), local PFS (**b**), distant PFS (**c**) and time to initiation of ADT or time to intensification of systemic therapy (**d**), after SBRT of osseous oligometastases of PC patients

The median follow-up after RT of oligometastases was 22.5 months (range 7.0–53.7 months). The median PSA-PFS was 6.9 months (range 1.1–28.4 months), see Fig. 1a. Imaging for detection of PSA-progression was conducted in 13 patients: nine ^68^Ga-PSMA-PET imaging, two ^11^C–Choline PET imaging, and two conventional CT scans. Local PFS (LPFS) after 2 years was 100%, see Fig. 1 b. One patient developed a local failure after 28.4 months. This patient sustained a local progression in the seventh thoracic vertebra in ^68^Ga-PSMA-PET imaging. The simultaneous staging by ^68^Ga-PSMA-PET showed the metastasis still to be a solitary metastasis. Due to the prior radiation to the spinal cord, no further RT was offered. ADT was initiated. This patient received 25 Gy to the whole vertebra and 40 Gy to the GTV in 5 fractions, resulting in an EQD2 of 100 Gy for α/β = 2 applied with TomoTherapy. All other patients with PSA progression and the aforementioned imaging showed distant failures (*n* = 12). Two patients with PSA failure received immediate ADT from their urologists without preceding imaging.

All patients showing a decrease in PSA level after RT of at least factor 10 (*n* = 6) later revealed a PSA-PFS of >12 months. Median PSA-PFS of this sub-group was 23.1 months (range 12.1–28.4 months).

Three patients are free from recurrence, one patient presented with a synchronous osseous metastasized PC, GS7, in the 4th lumbar vertebra after simultaneous primary hormone- and chemotherapy. Prostatectomy was conducted 4 weeks ago, the postoperative PSA level is lacking. The two other patients who are still free from recurrence show biochemical control >12 months, one with and one without ADT. The patient without ADT had a second SBRT in the iliac bone after the first SBRT in the pubic bone led to a PSA recurrence after 10.5 months, and is still free from PSA failure. Both patients have a PSA level below the detection limit. Patients in whom the PSA value has not even halved after RT (*n* = 5) showed only a short PSA-PFS of <12 months. Median PSA-PFS of this sub-group was 6.9 months (range 3.4–10.5 months). In four patients, the PSA level did not decrease after RT and systemic therapy were initiated or intensified.

Median Distant PFS (DPFS) was 7.36 months (range 1.74–54.34 months), see Fig. 1c. The time to initiation of ADT in patients treated without ADT was 9.3 months (range 2.6–36.1 months). In all patients, the time to intensification of systemic therapy or to initiation of ADT increased from 9.3 months to 12.3 months (range 2.6–36.1 months), see Fig. 1d. The three patients who benefitted most were the two patients with the metachronously occurring metastases (27.5 and 36.1 months) and one patient with synchronous two metastases (25.3 months). GS, ADT or the localization of the metastasis had no impact on PFS, the time to initiation of ADT, or intensification of systemic therapy.

**Fig. 2:**
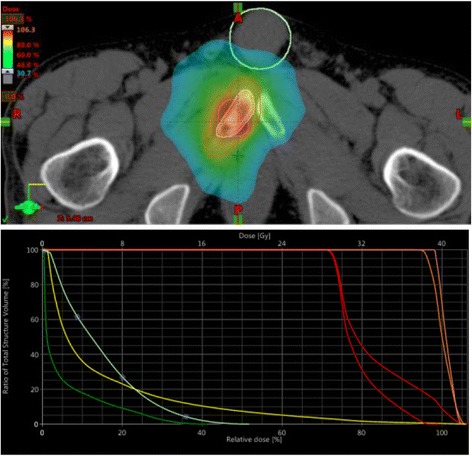
Dose distribution and Dose Volume Histogram of an SBRT (rapidarc) of the right pubic bone with 30 Gy to the gross target volume (GTV) + 2 cm (30 Gy-isodose encloses 85% of the PTV) and 40 Gy/median to the GTV as a simultaneously integrated boost (SIB) in 5 fractions

**Fig. 3:**
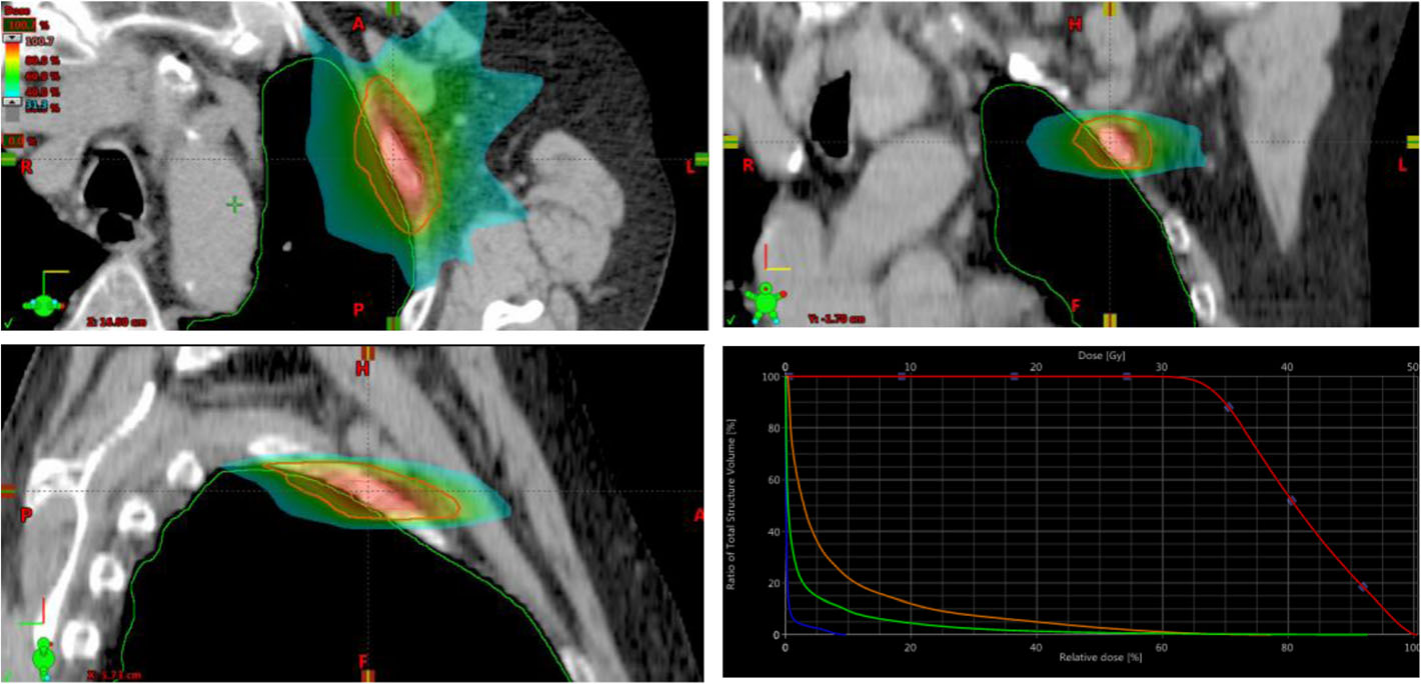
Dose distribution and Dose Volume Histogram of an SBRT (3D) of the second rib left with 30 Gy in 5 fractions (60%-isodose)

**Fig. 4:**
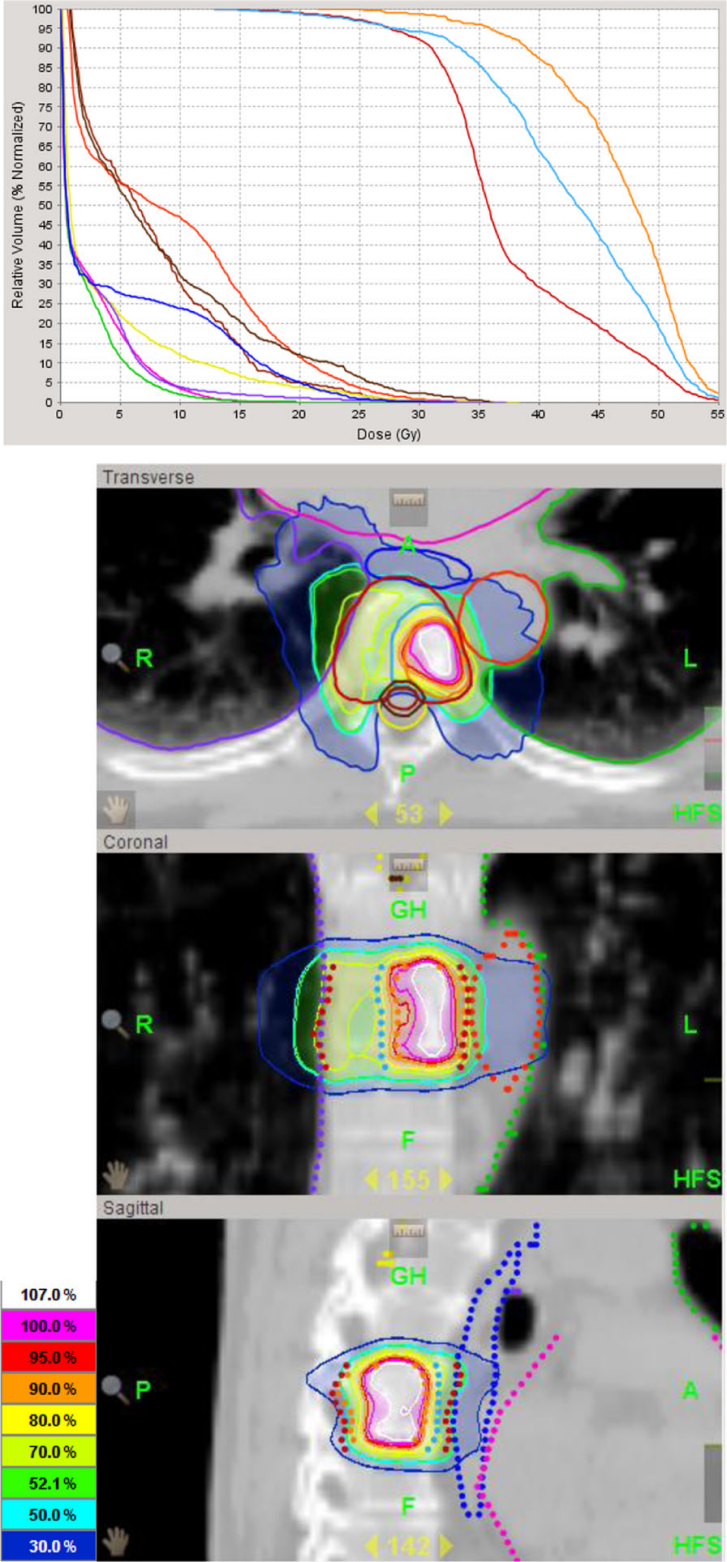
Dose distribution and Dose Volume Histogram of an SBRT of the thoracic vertebra 7 treated with tomotherapy for maximum sparing of myelon, oesophagus and thoracic aorta. The whole vertebra received 6 × 5 Gy (median) and the GTV (SIB) received 6 × 8 Gy (median). Of planned six fractions, only five were applied

Figures 2, 3 and 4 show some clinical examples of SBRT strategies. Figure 2 shows a rapidarc plan of an SBRT of the pubic bone, Fig. 3 illustrates a 3D planned SBRT of a rib metastasis and Fig. 4 shows a helical IMRT of a bone metastasis in a thoracic vertebra between the aorta and the myelon.

## Discussion

The aim of this study was to evaluate the impact of SBRT in oligometastasized PC patients. We evaluated 15 patients with 20 bone metastases cases undergoing locally ablative RT with a curative intent. We showed a high LCR with doses of EQD2 (equivalent dose in 2 Gy fractions) of around 100 Gy. We could demonstrate a beneficial risk-benefit profile with very low rates of side effects and high local control of the irradiated lesions.

Since oligometastasized PC patients represent a special subgroup of patients with long-term overall survival, locally ablative treatments seem justified especially to delay ADT and to spare patients from ADT-related side effects. As SBRT of individual metastases has shown a very good local control in several prospective and retrospective studies, Decaestecker et al. initiated a study comparing SBRT of ^11^C–choline PET-CT positive metastases to an active surveillance strategy [12]. The influence of the ablation of macroscopic metastases concerning further disease process will be investigated.

In the present evaluation, we found a median PSA-PFS of 6.9 months. Only in six cases, PSA failure was observed after 12 months, whilst in 14 cases PSA-failure was detected within the first 12 months. A single arm prospective study showed similar results, in which oligometastatic PC patients with up to three metastases were treated by a stereotactic RT [11]; after 2 years, only 18 out of 50 patients were tumor-free and seven other patients were treated one to three additional times with a stereotactic RT and then remained tumor-free until the end of the study. The median PFS was reported at 19 months; LCR was 100%. Given that distant metastasis occurred in 31 patients, it could reasonably be assumed that most patients were understaged, and thus that patients already were already at least microscopically polymetastasized at the beginning of RT.

We found that all patients showing a decrease of PSA level after RT of at least factor 10 (*n* = 6) revealed a PSA-PFS of >12 months. The sub-group of patients whose PSA level dropped by more than a tenfold had a median PSA-PFS of 23.1 months. In contrast, patients whose PSA level had not even halved after RT showed a median PSA-PFS of only 6.7 months. Post-RT PSA levels either dropped by more than a tenfold, dropped to less than half, or continued to increase directly after treatment. This suggests a correlation between the diminution of PSA levels after SBRT and prolonged recurrence-free survival. Given the small study cohort, these considerations should be observed with caution.

Three of our 15 patients had concomitant ADT. Due to the fact that the start of ADT was not consistent within the patient cohort, the PSA-PFS data should be interpreted with caution. The combination of a locally ablative RT to the visible lesions with concomitant ADT was studied in a prospective single-arm Spanish-Swiss study [22]; in 50 patients, a PFS of 54%, a clinical PFS of 58% and an OS of 92% after a median time period of 30 months was achieved. ADT was not standardized and was given for a median time of 12 months (range 3–34 months). The ideal duration of ADT is frequently debated, in primary as well as in an adjuvant RT setting. One cannot directly compare the indication for ADT between the primary and oligometastasized situation. However, if we believe in the concept of an oligometastasized stage, an approach analog the primary treatment situation appears to be justified given the fact that there is a local problem with one known separate accumulation of tumor cells. Jones et al. have demonstrated an absolute survival benefit of 4% at 10 years for the combination of a four-month ADT and RT of the prostate compared with RT alone [23]. Importantly, distant metastases occurred significantly less frequently in the ADT group. A short-term ADT is apparently sufficient for the treatment of subclinical metastasis in patients with intermediate-risk PC. In contrast, in the high- and very high-risk situation, long-term ADT of least 24 months seems to be superior to short-term ADT [24]. Not only is the effectiveness of RT with or without ADT proven, but also the importance of local ablative therapy with sustained ADT. Widmark et al. and later Warde et al. described a significant OS benefit for the combination of RT and lifelong ADT compared to ADT alone [25, 26]. They concluded that local therapy improves the efficacy of androgen suppression significantly. Whether this conclusion applies to the oligometastatic situation has not yet been evaluated prospectively. We found no difference between the groups with and without ADT-related to PSA-PFS and the time to initiation of ADT or intensification of systemic therapy, however, our patient cohort is small.

Biologically, oligometastases are defined as a state in which the patient shows distant relapse in only a limited number of regions. In this case, the patient may benefit from a local tumoricidal treatment of all noticeable lesions. However, many patients with an initial oligometastasized stage develop more metastases and progress to a polymetastasized stage. To improve the patient selection for the local therapy in oligometastasized patients vs. systemic therapy in polymetastasized patients, biological predictors of progression are needed.

The limited effectiveness of treatments of oligometastases may be due to inability to recognize all present metastatic lesions and the situation might be staged as oligometastatic when in reality it is already widespread cancer [27]. There is a second group of oligometastasized patients in addition to the aforementioned patient group as ADT or other systemic therapies become more widely applicable. These are patients who had widespread metastases that were mostly eradicated by systemic agents. Systemic therapy can fail to destroy tumor cells, for example, due to the presence of drug-resistant cells or due to the tumor site. Thus, effective chemotherapy may fail to be curative because of only a few metastases. This example highlights the importance of markers specifically related to where the malignant cells are located. Oligometastasized and poly-metastasized tumors require different therapy schemes. New techniques of RT may allow curative treatment of such oligometastases either alone or in combination with systemic therapy. Their effectiveness will be critically dependent on the specificity and sensitivity of tumor imaging.

Conclusions should be drawn with caution because the presented study has some limitations. The analyzed patient group was very small, the follow-up is rather small and the study has a retrospective nature. Therefore, publication of all treatment concepts with an SBRT approach seem appreciated to give treating physicians as much information as possible to treat these patients adequately. Additionally, cases should be encompassed in a multi-institutional pooled analysis to confirm our results based on larger patient numbers.

## Conclusion

We could show that SBRT of bone metastases is a highly effective therapy with an excellent risk-benefit profile. However, PFS was limited due to a high distant failure rate, implying the difficulty for patient selection for this oligometastatic concept. Research on biomarkers besides PSA identifying purely oligometastasized patients would be of great benefit. SBRT offers high local cancer control rates in bone oligometastases of PC and should be evaluated with the aim of curation or to delay modification of systemic treatment.

## Abbreviations

ADT: Androgen deprivation therapy
CTV: Clinical target volume
DPFS: Distant progression-free survival
GTV: Gross tumor volume
LCR: Local control rate
LPFS: Local progression-free survival
PC: Prostate cancer
PET: Positron emission tomography
PSA: Prostate specific antigen
PSA-PFS: PSA progression-free survival
PTV: Planning target volume
RT: Radiotherapy
SBRT: Stereotactic body radiotherapy
